# Supplementary material for: The Associations between Upper and Lower Body Muscle Strength and Diabetes among Midlife Women
Source: Int J Environ Res Public Health. 2022 Oct 21;19(20):13654. doi: 10.3390/ijerph192013654 (PMC9602555; doi:10.3390/ijerph192013654)
Supplement: Supplementary file 1 [file ijerph-19-13654-s001.zip › ijerph-1959149-supplementary.pdf]

Supplementary Table S1. Characteristic differences between included and excluded participants

Characteristics	Total N (n=1201)	Included (n=1170, 97.4%)	Excluded (n=31, 2.6%)	P-value
Self-reported measures, n (%)				
Age, years (mean ± sd)				0.276
45-54 (50.5 ± 2.7)	525 (43.7)	511 (43.7)	14 (45.2)	
55-64 (59.1 ± 2.9)	528 (44.0)	512 (43.8)	16 (51.6)	
65-69 (66.7 ± 1.3)	148 (12.3)	147 (12.6)	1 (3.2)	
Ethnicity				0.418
Chinese	974 (84.0)	948 (83.8)	26 (92.9)	
Malay	66 (5.7)	65 (5.6)	1 (3.6)	
Indian	119 (10.3)	118 (10.1)	1 (3.6)	
Education level				0.404
Primary level or below	172 (14.5)	170 (14.7)	2 (6.9)	
Pre-university	775 (65.3)	753 (65.1)	22 (75.9)	
University	239 (20.2)	234 (20.2)	5 (17.2)	
Housing type				0.336
Public (1-3 room)	144 (12.1)	138 (11.9)	6 (20.7)	
Public (4-5 room)	806 (67.7)	789 (68.0)	17 (58.6)	
Private	240 (20.2)	234 (20.2)	6 (20.7)	
Parity				0.025
Nulliparous	207 (17.2)	197 (16.8)	10 (32.3)	
Multiparous	994 (82.8)	973 (83.2)	21 (67.7)	
Menopausal status				0.996
Premenopausal	151 (12.6)	147 (12.6)	4 (12.9)	
Perimenopausal	189 (15.7)	184 (15.7)	5 (16.1)	
Postmenopausal	861 (71.7)	839 (71.7)	22 (71.0)	
Smoking status				0.442
Non-smokers	1170 (97.9)	1143 (97.9)	27 (100.0)	
Smokers	25 (2.1)	25 (2.1)	0 (0.0)	
Alcohol consumption				0.848
Non-alcohol consumers	1153 (96.8)	1128 (96.8)	25 (96.2)	
Alcohol consumers	38 (3.2)	37 (3.2)	1 (3.8)	
Physical activity				0.176
Yes	730 (61.4)	708 (61.1)	22 (73.3)	
No	458 (38.6)	450 (38.9)	8 (26.7)	
Objectively measured measures, n (%)				
Height (m)				0.385
<1.55	430 (35.8)	418 (35.7)	12 (38.7)	
1.55-1.60	481 (40.0)	472 (40.3)	9 (29.0)	
>1.60	290 (24.1)	280 (23.9)	10 (32.3)	
BMI (kg/m^2)				0.756
Underweight (<18.5)	61 (5.1)	59 (5.0)	2 (6.5)	
Normal (18.5-22.9)	497 (41.4)	482 (41.2)	15 (48.4)	
Overweight (23.0-27.5)	413 (34.4)	403 (34.4)	10 (32.3)	
Obese (>27.5)	230 (19.2)	226 (19.3)	4 (12.9)	
Visceral adipose tissue (cm^2)				0.647
<88.6	400 (33.6)	389 (33.5)	11 (35.5)	

88.6-131.0	395 (33.1)	383 (33.0)	12 (38.7)	
>131.0	397 (33.3)	389 (33.5)	8 (25.8)	
ALM/height[2]				0.984
< 5.4	622 (51.8)	606 (51.8)	16 (51.6)	
≥ 5.4	579 (48.2)	564 (48.2)	15 (48.4)	
Handgrip strength (kg)				0.182
<18	269 (22.4)	259 (22.1)	10 (32.3)	
≥18	932 (77.6)	911 (77.9)	21 (67.7)	
5-repetition chair stand test (s)[1]				0.363
≥12	522 (44.2)	519 (44.4)	3 (30.0)	
<12	658 (55.8)	651 (55.6)	7 (70.0)	
Muscle strength index				
Poor[2]	154 (12.9)	153 (13.1)	1 (4.5)	0.001
Intermediate[3]	474 (39.8)	472 (40.3)	2 (9.1)	
Normal[4]	564 (47.3)	545 (46.6)	19 (86.4)	

Missing data accounted for up to 3.5% of overall data.

[1] 5-repetition chair stand test: Time taken to rise from a seated position and back down to a sitting position five times.

[2] Poor MSI: HGS < 18 kg and RCS ≥ 12 s.

[3] Intermediate MSI: HGS < 18 kg or RCS ≥ 12 s.

[4] Normal MSI: HGS ≥ 18 kg and RCS < 12 s.
